# SNooPer: a machine learning-based method for somatic variant identification from low-pass next-generation sequencing

**DOI:** 10.1186/s12864-016-3281-2

**Published:** 2016-11-14

**Authors:** Jean-François Spinella, Pamela Mehanna, Ramon Vidal, Virginie Saillour, Pauline Cassart, Chantal Richer, Manon Ouimet, Jasmine Healy, Daniel Sinnett

**Affiliations:** 1 CHU Sainte-Justine Research Center, Université de Montréal, Montreal, QC Canada; 2 Department of Pediatrics, Faculty of Medicine, Université de Montréal, Montreal, QC Canada; 3 Division of Hematology-Oncology, CHU Sainte-Justine Research Center, 3175 Côte Sainte-Catherine, Montreal, QC H3T 1C5 Canada

**Keywords:** Somatic variant, Low-pass next-generation sequencing, Machine learning, Random Forest

## Abstract

**Background:**

Next-generation sequencing (NGS) allows unbiased, in-depth interrogation of cancer genomes. Many somatic variant callers have been developed yet accurate ascertainment of somatic variants remains a considerable challenge as evidenced by the varying mutation call rates and low concordance among callers. Statistical model-based algorithms that are currently available perform well under ideal scenarios, such as high sequencing depth, homogeneous tumor samples, high somatic variant allele frequency (VAF), but show limited performance with sub-optimal data such as low-pass whole-exome/genome sequencing data. While the goal of any cancer sequencing project is to identify a relevant, and limited, set of somatic variants for further sequence/functional validation, the inherently complex nature of cancer genomes combined with technical issues directly related to sequencing and alignment can affect either the specificity and/or sensitivity of most callers.

**Results:**

For these reasons, we developed SNooPer, a versatile machine learning approach that uses Random Forest classification models to accurately call somatic variants in low-depth sequencing data. SNooPer uses a subset of variant positions from the sequencing output for which the class, true variation or sequencing error, is known to train the data-specific model. Here, using a real dataset of 40 childhood acute lymphoblastic leukemia patients, we show how the SNooPer algorithm is not affected by low coverage or low VAFs, and can be used to reduce overall sequencing costs while maintaining high specificity and sensitivity to somatic variant calling. When compared to three benchmarked somatic callers, SNooPer demonstrated the best overall performance.

**Conclusions:**

While the goal of any cancer sequencing project is to identify a relevant, and limited, set of somatic variants for further sequence/functional validation, the inherently complex nature of cancer genomes combined with technical issues directly related to sequencing and alignment can affect either the specificity and/or sensitivity of most callers. The flexibility of SNooPer’s random forest protects against technical bias and systematic errors, and is appealing in that it does not rely on user-defined parameters. The code and user guide can be downloaded at https://sourceforge.net/projects/snooper/.

**Electronic supplementary material:**

The online version of this article (doi:10.1186/s12864-016-3281-2) contains supplementary material, which is available to authorized users.

## Background

The advent of next-generation sequencing (NGS) has allowed unbiased in-depth interrogation of cancer genomes and has led to the identification of a number of tumor-specific mutations responsible for driving oncogenesis in multiple cancer types including skin carcinoma [1, 2], bladder cancer [3], prostate cancer [4, 5], colorectal cancer [6], breast cancer [7–12], medulloblastoma [13] and leukemias/lymphomas [14–18]. Sequencing of matched normal-tumor pairs is routine in cancer research in order to identify a relevant, and limited, set of somatic variants for further functional validation. However, the inherently complex nature of cancer genomes [19], the heterogeneity of tumor samples, as well as random (or systematic) sequencing and alignment errors can affect the specificity and/or sensitivity of most variant callers [20]. Of particular interest is the identification of low-frequency tumor alleles that arise in subclonal tumor cell populations, often contributing to treatment failure and relapse [21–25]. While NGS provides the opportunity to track specific mutations in tumor subclones and potentially uncover mutations with relapse driving potential [26], the identification of such mutations within the primary tumor cell population is often confounded and difficult to distinguish from background noise, as evidenced by the consistently low concordance rates between algorithms [20].

A number of methods have been developed to overcome these challenges in somatic mutation calling in matched normal-tumor samples. These methods are either heuristic, such as VarScan2 [27] that relies on independent analysis of tumor and normal genomes followed by a statistical Fisher’s Exact Test of read counts for variant detection, or probabilistic, such as SomaticSniper [28], JointSNVMix [29], Strelka [30] and MuTect [31] that use Bayesian modeling to estimate likely joint normal-tumor genotype probabilities. Yet most somatic variant callers still perform poorly at low sequencing depths [32]. Indeed, large investments in validation efforts are needed to compensate the high false positive rates of most exploratory projects that are aimed at investigating more than a small set of top ranked high confidence somatic variants. And though progressively larger cohorts of individuals are being sequenced, the tendency towards shallow or low-coverage data is still de rigueur, particularly for whole genome sequencing initiatives, due to high sequencing costs.

To address these issues, we developed SNooPer, a versatile data mining approach that uses Random Forest (RF) classification [33] to accurately identify somatic variants in complex, low-depth sequencing data. Unlike available somatic variant callers, SNooPer does not rely on user-defined parameters but builds upon the data itself to construct powerful prediction models and increase calling performances. Using both simulated and real datasets, we evaluated SNooPer’s ability to detect true somatic mutations in unbalanced, low-depth datasets while limiting false positive calls, and compared its performance to three benchmarked algorithms - Varscan2 [27], JointSNVmix [29], and MuTect [31].

## Design and implementation

### Design

The purpose of SNooPer is to distinguish sequencing errors (false positives - FPs) from actual somatic variants (true positives - TPs) in matched normal-tumor sequencing data. SNooPer uses a Leo Breiman RF classifier [33] which was chosen because of its limited tendency to overfit training data [33], its efficient management of very large datasets and its capability to cope with unbalanced datasets, in which one class (in this case sequencing error) is overrepresented in comparison to the other (somatic variation). RF applies bootstrap aggregation or “bagging” (subsets of the training data are selected with replacement) on multiple decision trees grown without pruning in which each node is split based on the information provided by a subset of randomly selected features. For each variant position, 15 features expected to be informative for the identification of true somatic mutations are extracted and/or calculated from the mpileup files. The complete list of features and their descriptions are presented in Additional file 1: Table S1). These features are divided into five main groups: i) quality bias of alternative bases (related to base and mapping phred quality values), ii) coverage and VAF, iii) location along the read, iv) strand bias, and v) others. When appropriate, features are evaluated with respect to reference bases at the same position (vs_ref). To reduce over-fitting on training data and when possible, instead of absolute values, features are normalized using the corresponding median value calculated from randomly extracted subsets of variants from corresponding mpileup files (vs_med). For each model, features are ranked and selected by measuring information gain (IG) or Kullback–Leibler divergence [34] with respect to the class (InfoGainAttributeEval method, Weka suite [35], Additional file 2). Given that the relative importance of features for prediction may vary depending on the dataset or the genomic region of interest, the flexibility of SNooPer allows a new set of features to be selected in the training of each new model. By default, during each training phase and using the remaining bootstrap datasets (unused portion of the bootstrap as a test set), RF estimates the generalization error using the out-of-bag (oob) error as an internal control. Once trained, the model is saved and applicable to any new dataset presented to SNooPer. In the event that validation subsets are not readily available, we have also developed a series of pre-trained classification models that can be used to call variants from most datasets, including those obtained from other cancer types.

### Code implementation

SNooPer is written in the Perl programming language and has a few dependencies: Math::CDF, Text::NSP::Measures::2D::Fisher, Statistics::Test::WilcoxonRankSum and Statistics::R. Furthermore, SNooPer uses a RF classifier implemented in Weka suite (3.6.10 or greater) [35] and requires the Java Runtime Environment (1.5 or greater). Additional and optional filters (germline dataset and blacklisted genomic regions) require a Bedtools intersect function [36]. Receiver Operating Characteristic (ROC) and Precision-Recall (PR) curves are drawn using the R package ‘pracma’ (Practical Numerical Math Functions). Detailed information about how to install and run SNooPer, including all available options, are described in the Additional file 2.

### Workflow

**Fig. 1:**
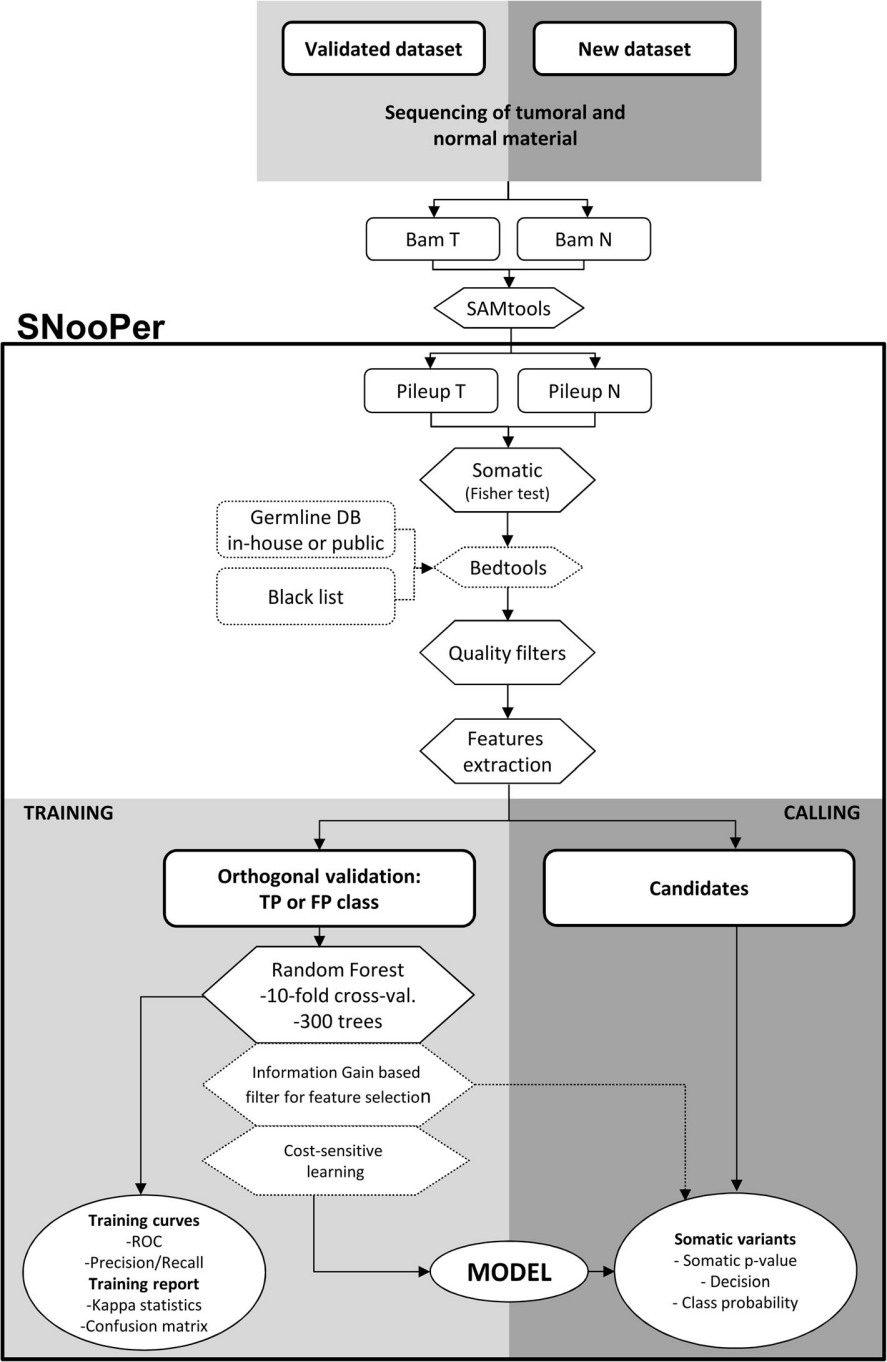
Workflow of SNooPer’s algorithm. SNooPer uses both normal and tumor files in a SAMtools mpileup format as input. It requires a training phase in which an orthogonal validation (re-sequencing) dataset is used to train the RF classification model that is subsequently used to call somatic variations in the test dataset. Light grey boxes represent the training steps while dark grey boxes represent calling steps. Dotted boxes represent optional steps in the workflow. Circles represent the output following either the training or calling phases

The complete workflow of SNooPer's algorithm is shown in Fig. 1 (common keywords between the figure and the following description are indicated in italics).

#### Somatic testing and feature extraction

SNooPer expects both normal and tumor files in SAMtools mpileup format (*Pileup T* vs. *Pileup N* in Fig. 1). To call variants as somatic, a *Fisher's exact test* is applied to compare the distribution of reads supporting the reference and the alternative allele between normal and tumor samples. Optionally, SNooPer can integrate two additional filters input as BED format files (*Bedtools* step in Fig. 1) to exclude overlaps with any provided germline dataset (e.g. common polymorphisms from 1000 Genomes dataset [37]) or blacklisted genomic regions (e.g. poorly mappable regions from the RepeatMasker sequence [38]). Using the default parameters of *quality filters*, the algorithm only considers positions presenting at least one read (mapping quality value - MQV ≥10) supporting the alternative allele (base quality value - BQV ≥20), and requires a minimum coverage of 8X in both the tumor sample and its normal counterpart. *Features extraction* (S1 Table) is then make for each putative somatic variants that passed these filters.

#### Training phase

During this phase, identified variants are divided into two classes according to *orthogonal validations*: a false positive class (errors) and a true positive class (validated variations). This dataset is then used to train the *RF classifier*. To improve time-effectiveness, the default number of trees used to build the model is limited to 300 (see Results). At each node, Log2(total number of attributes) + 1 features are randomly selected. The oob error rate is used as an unbiased estimate of the classification error as trees are added to the forest during training. The classification error rate is also controlled by default using a *10-fold cross-validation* estimator. Informative features for the classification are selected by measuring *information gain* or Kullback–Leibler divergence. ROC and PR curves (*Training curves*) and the related Areas Under the Curves (AUCs) are calculated for each training run (Additional file 3). Furthermore, SNooPer was designed to allow variable VAF intervals for targeted training as well as *cost-sensitive learning* to compensate unbalanced data and allow for high sequencing error rates. For discovery, users can also vary the cost of false negatives and false positives to reflect more liberal or conservative modeling. The trained *model* can be saved and applied to any subsequent dataset.

#### Calling phase

During the calling phase, the trained *model* as well as new tumor and matched normal mpileup files are used as input. A *Fisher’s Exact Test* is performed (*Pileup T* vs. *Pileup N*) to identify putative somatic variants. Features that have been used to train the model of interest are calculated from the mpileup files for each of the putative variants and the *model* is applied for classification. The calling phase outputs a VCF file, which includes the somatic p-value from the Fisher’s exact test, a categorical annotation of prediction (“PASS” or “REJ”) and associated class probability (from 0.5 to 1) for each somatic variant identified, allowing the user to adjust numerical filters with more flexibility than that allowed by categorical predictions.

SNooPer’s run-time efficiency is acceptable. For example, to run an entire training phase using 250 TPs and 30,000 FPs from 4 sets of whole-exome sequencing (WES) data as input (12 matched normal-tumor pileup pairs) and 300 trees and a 10-folds cross validation as training parameters, the algorithm runs for about 8 h on a standard 12-core computer workstation with 24 Gbytes of memory, each core running at 2.667 Ghz. The time taken by the Random Forest increased linearly with the number of trees built: 0.58, 8.43, 24.45, 50.45 and 83.22 min were needed to build 10, 100, 300, 600 and 1,000 trees, respectively (these periods of time excluded the time taken for the calculation of features which relies on the size of the training dataset, not on the the number of trees used). Finally, during a standard calling phase, using a single-core (2.667 Ghz), SNooPer analyses approximately 5,000 pileup lines per minute.

## Results and discussion

### Classifier performance assessment

**Fig. 2:**
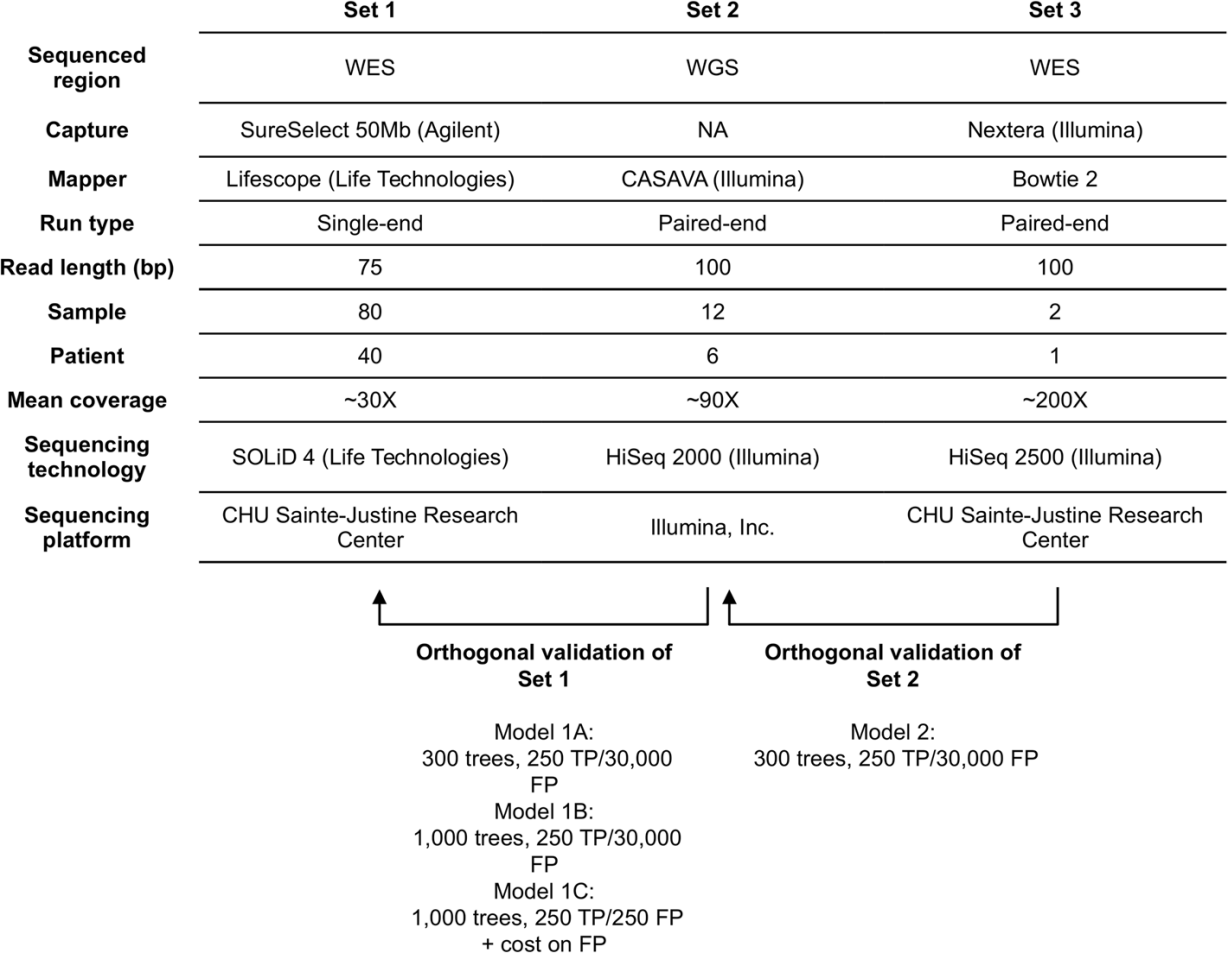
Datasets used to develop and assess SNooPer. All 3 datasets were generated from real childhood acute lymphoblastic leukemia samples. Arrows indicate sequencing overlaps between datasets. Re-sequencing was used as orthogonal validation for the training phases of the algorithm. RF Models (1A, 1B, 1C and 2) resulting from these training phases are shown below the corresponding arrows

For the development and assessment of SNooPer, we used a series of real NGS datasets from 40 unrelated childhood acute lymphoblastic leukemia (cALL) patients (Fig. 2). All study subjects were French-Canadians of European descent from the established Quebec cALL (QcALL) cohort [39]. For each patient, bone marrow and blood samples were collected at diagnosis prior to treatment (patient tumor) and at remission (matched patient normal). DNA was extracted using standard protocols [40] and sequenced on the Life Technologies SOLiD 4 System to constitute Dataset 1 (mean coverage on targeted region =30X). 12 cALL patient genomes (6 tumor-normal), overlapping Dataset 1, were also sequenced by Illumina, Inc. on the HiSeq 2000 (mean coverage =90X) and considered as orthogonal validation for Dataset 1. Finally, 2 samples sequenced at higher depth on the Illumina system (HiSeq 2500, mean coverage of 200X), overlapping Datasets 1 and 2, were used as validation for Dataset 2 (Fig. 2 and Additional file 2 for details). To test our somatic caller, we generated 4 model scenarios constructed using these 3 datasets (Fig. 2 and Additional file 2 for details). These scenarios were constructed to test the effect on training of the RF classifier of either a variation of the number of trees (models 1A vs 1B), of the skewness of the (unbalanced) datasets (models 1B vs 1C), or of the sequencing depth and technology (model 2 vs model 1A).

#### Classifier training

**Fig. 3:**
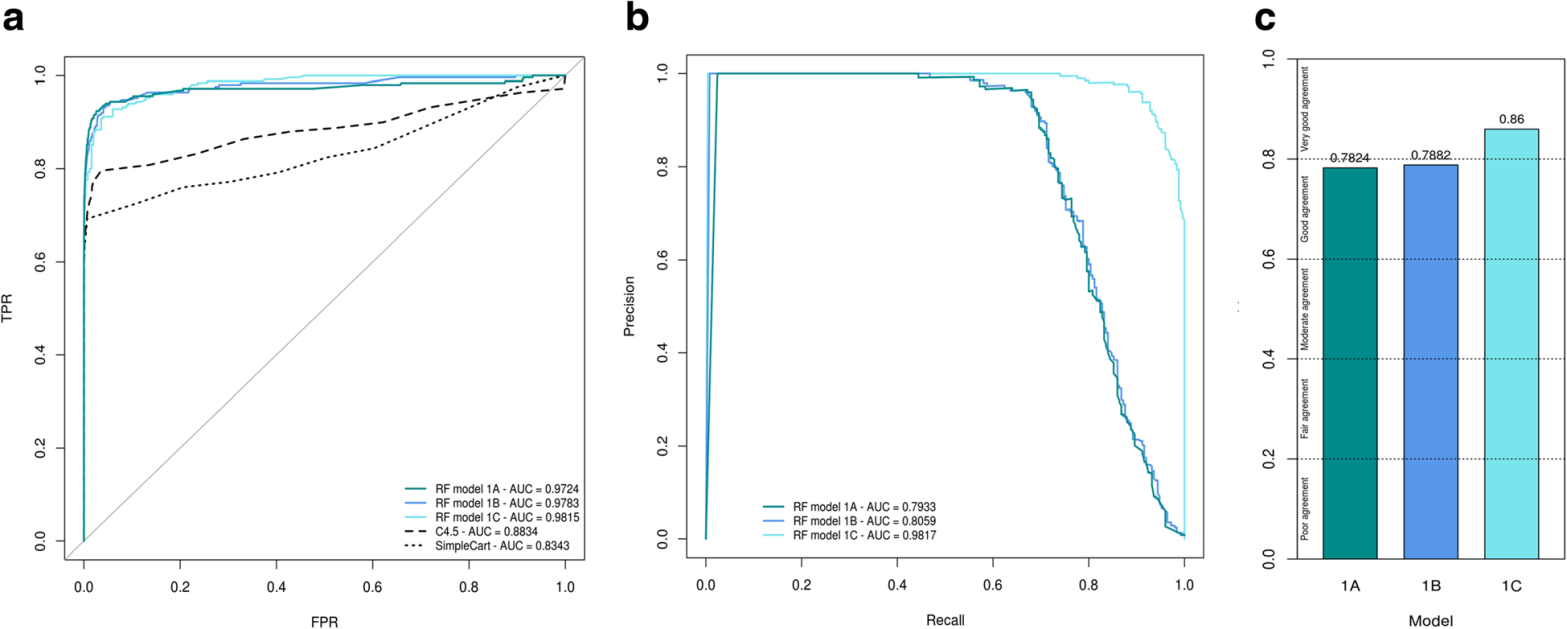
Training assessment of Model 1A, 1B and 1C. Data used to construct these curves were obtained from SNooPer’s RF training phase using Dataset 2 as a validation set and a subset of Dataset 1 as training set. *Dark cyan*, *blue* and li*ght blue* represent SNooPer's Model 1A, 1B and 1C, respectively and AUCs are shown for each model. **a** ROC curves. Solid, dashed and dotted lines represent RF, C4.5 (J48) and SimpleCart algorithms respectively. TPR stands for True Positive Rate and FPR for False Positive Rate. **b** PR curves. **c** Cohen's Kappa coefficient

**Fig. 4:**
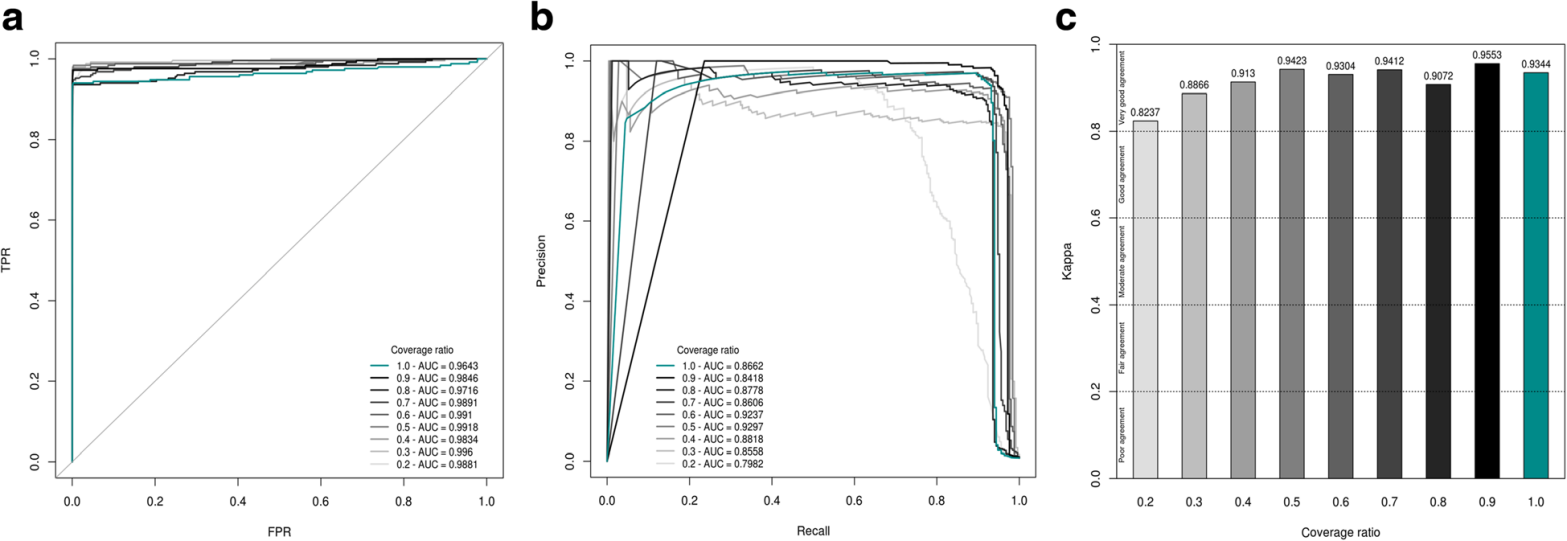
Training assessment of Model 2. The data used to construct these curves were obtained from training phases using Dataset 3 as validation set and either the original Dataset 2 (*dark cyan*) or an artificial version of Dataset 2 (*shades of grey*) in which the coverage was gradually subsampled from 10% (ratio of 0.9 ~ 81X; *darkest grey*) to 80% (ratio of 0.2 ~ 18X; *lightest grey*) as training set. AUCs are shown for each model. **a** ROC curves. Solid, dashed and dotted lines represent RF, C4.5 (J48) and SimpleCart algorithms respectively. TPR stands for True Positive Rate and FPR for False Positive Rate. **b** PR curves. **c** Cohen’s Kappa coefficient

10-fold cross-validation was used to compare the performance of SNooPer’s classification based on the training for each Model (Fig. 2, Additional file 2). ROC and PR curves were generated and the related AUC was measured on each training dataset (Figs. 3a, b and 4a, b). Cohen’s kappa coefficient [41] was also used to assess the performances of SNooPer’s RFs under each modeled condition (Figs. 3c and 4c). To assess SNooPer’s ability to classify an unbalanced test set while being trained with a reduced and balanced training dataset, we constructed Model 1C using 250 true and false positive calls from the training set. To cope with the unbalanced test set on which the model was applied, we weighted training instances (stronger cost on false positives) using SNooPer’s cost sensitive training option.

Evaluation of the oob error rates for Models 1A, 1B, and 1C (0.003, 0.003 and 0.022, respectively), suggested powerful classification performances for SNooPer’s RF. ROC AUCs (0.9724, 0.9783 and 0.9815), PR AUCs (0.7933, 0.8059 and 0.9817) and Kappa coefficients (0.7824, 0.7882 and 0.8600) also showed good agreement for SNooPer’s RF under Models 1A, 1B, and 1C, respectively. Improved training statistics for Model 1C were due to a strong reduction of the number of false positives in the training dataset from 30,000 to 250.

We compared classification performances of SNooPer’s RF to two other decision tree generators: we trained Dataset 1 using the C4.5 algorithm [42] (J48 in Weka suite) and SimpleCart [35]. For C4.5 classification, a confidence factor of 0.25 was used for pruning and we set a minimum of two instances per leaf. For SimpleCart, a minimum number of two observations at the terminal nodes was used with 5-fold internal cross-validation. C4.5 and SimpleCart trainings clearly underperformed RF with ROC AUCs of 0.8834 and 0.8343 respectively (Fig. 3a).

To investigate how coverage, sequencing technologies and post-sequencing data processing (e.g. mapping method) may influence SNooPer’s performance, we constructed Model 2 using Dataset 2. The training phase for this Illumina whole-genome sequencing (WGS) dataset (mean coverage of 90X) returned a oob of 0.001, a Kappa coefficient of 0.9344 and ROC and PR AUCs of 0.9643 and 0.8662.

Firstly, to evaluate the influence of coverage on SNooPer’s classification performances, we constructed artificial test datasets. The coverage of Dataset 2 was reduced by 10% (~81X) to 80% (~18X), through subsampling using SAMtools [43]. We found no clear decrease in performance except at sequencing depths below 18X (80% reduction) as illustrated by a PR AUC of 0.7982 and a Kappa coefficient of 0.8237 (Fig. 4). Interestingly, the best overall performance was observed at 45X (50% reduction) with ROC and PR AUCs of 0.9918 (2nd best) and 0.9297 (best) and a Kappa coefficient of 0.9423 (2nd best). At ~36X (40% reduction in coverage), SNooPer performances were better than those obtained for Dataset 1 (mean 30X depth coverage) with ROC, PR AUCs and a Kappa coefficient of 0.9834, 0.8818 and 0.9130 compared to 0.9724, 0.7933 and 0.7824 obtained using Model 1A on Dataset 1. The improved performance is likely due to differences in sequencing and post-sequencing data processing methods, suggesting that inherent sequencing platform and/or mapping biases can influence SNooPer’s classification. Overall, and despite slight variations between Datasets 1 and 2, evaluation of the performance of the classification model yielded satisfying results across distinct datasets and sequencing technologies, further highlighting the flexibility of SNooPer’s classification model.

#### Comparison with other methods

To achieve an accurate and unbiased estimate of the performance of SNooPer in predicting somatic variants, and to compare SNooPer to other routinely used somatic single nucleotide variant (SNV) callers including Varscan2 255 [27], JointSNVMix [29] and MuTect [31] (Additional file 2), we randomly excluded whole exome sequencing data from Dataset 1 before training and used it as test set (Fig. 2, Additional file 2). This test set is a particularly demanding dataset given its severely unbalanced class distribution, with approximately 1 true somatic variation per million false positives presenting at least one supporting read (TP/FP =9.3E-07).

**Fig. 5:**
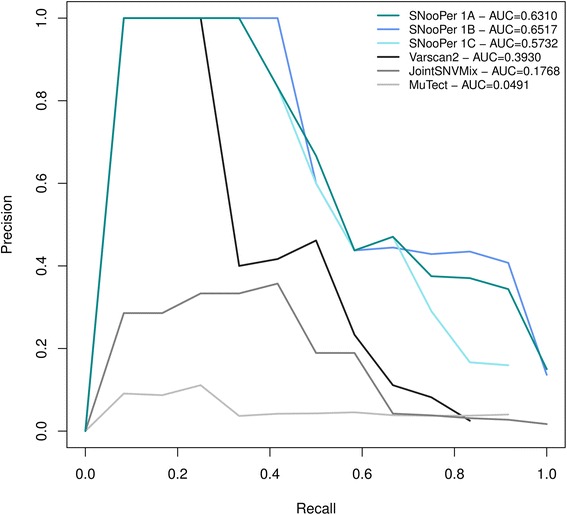
Precision – Recall curves for method comparison. Data used to construct these curves were obtained from SNooPer’s calling phase using Model 1A (*dark cyan*), Model 1B (*blue*), Model 1C (li*ght blue*), Varscan2 (*black*), JointSNVMix (dark grey) and MuTect (light grey) on the test set. The test set was built using a subset of Dataset 1 that was kept completely separate during the training phase. AUCs are shown for each model

To accurately compare the performances of different algorithms, recall values were fixed for all callers and we estimated the precision (fraction of real calls) of each algorithm on the test dataset. Data were filtered on numerical values for all callers instead of on categorical variables only (Additional file 2). To evaluate the predictive performance of each somatic SNV calling algorithm, we generated PR curves and assessed the related AUCs (Fig. 5). Regardless of the trained model used, SNooPer outperformed all other callers on this test dataset. The lowest AUC obtained for SNooPer (0.5732) was obtained using Model 1C while JointSNVMix, Varscan2 and MuTect reached AUCs of 0.3930, 0.1768, and 0.0491 respectively. SNooPer Models 1A and 1B, trained using 300 and 1,000 trees respectively, showed very similar performances with AUCs of 0.6310 and 0.6517. For Model 1C, reweighting of false positives correctly compensated the bias that was generated from the use of a balanced training set that was not representative of the test set. Overall, the use of SNooPer’s RF classification algorithm lead to efficient identification of clonal and subclonal somatic variations with VAFs ranging from 0.16 to 0.58, with low false discovery rates of 0.363, 0.342 and 0.367 for Models 1A, 1B and 1C, respectively (mean false discovery rate - FDR over tested points). Among the 3 models, only Model 1C missed a mutation with low VAF (0.16). Limited performances and high mean FDRs observed for other methods (FDR_Varscan2_ = 0.527, FDR_JointSNVMix_ =0.822, FDR_MuTect_ =0.945) were probably due to the suboptimal quality of SOLiD sequencing data with high sequencing/mapping error rates and low coverage (mean coverage of ~30X) for the callers’ standards. More specifically, given the limited power of the strand bias feature to discriminate true positive calls from errors in Dataset 1 (see Additional file 1 and Feature Selection section in Additional file 2), methods such as Varscan2 and MuTect that rely significantly on this feature to call variations were expected to underperform on these data. Varscan2 filters out variants with >90% of the supporting reads originating from the same strand, and MuTect applies a restrictive strand bias filter based on a separate calling step on each strand implemented to avoid variants supported by a biased alignment. As expected, SNVs that were missed by these two algorithms were all positions that were affected by strand bias. Still, despite its strong strand bias filter, Varscan2 showed the best overall performance of the three benchmarked algorithms that were tested here. On the other hand, MuTect is known to be a very sensitive SNV caller that is powered to detect low VAF mutations. However, as illustrated in Additional file 4: Figure S2, the VAF distribution of somatic MuTect variations was clearly skewed toward very low VAFs compared to the distribution of true somatic SNVs present in the test set, leading to a large number of false positives in the MuTect output. A similar pattern with an increase in low VAF calls (<0.2) was observed for JointSNVmix, also resulting in increased false positive calls. Unlike other callers, the SNooPer algorithm involves a training phase where class assignment is directly learned from the dataset at hand, and this translated into a VAF distribution that matched the true positives distribution. Moreover, under Models 1A and 1B, SNooPer identified less than 90 somatic SNVs that included all true somatic SNVs present in the test dataset, while MuTect (power ≥0.16) identified 274 somatic variants, Varscan2 (somatic *p*-value ≤0.17) 397, and JointSNVMix (P(somatic) ≥0.29) identified 705 somatic SNVs, which included 92%, 83% and 100% of the true somatic variants, respectively. SNooPer's somatic SNV calls under Model 1A and 1B were thus more precise and no true somatic variants were missed, further highlighting its superior performance. With a higher sensitivity and specificity for somatic SNV detection in our low quality test set (mean coverage <30X), SNooPer outperformed commonly used somatic variant callers such as Varscan2, JointSNVMix and MuTect. Importantly, this report was not meant to question the performance of benchmarked callers that have proven to be efficient and that classically show satisfactory results with high coverage datasets.

#### Real data analysis

**Fig. 6:**
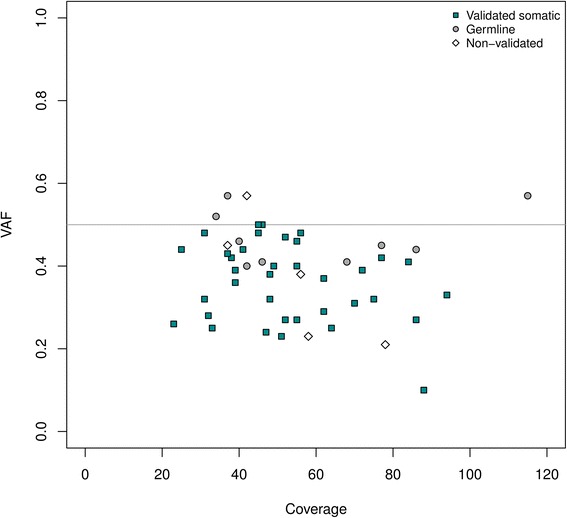
Validation plot. Distribution of 50 randomly selected SNVs called using SNooPer's Model 1A on the independent validation set constituted of samples obtained from 34 childhood acute lymphoblastic leukemia patients (matched normal and tumor). All selected SNVs were heterozygous with a VAF < 0.6, predicted as damaging (Sift [42 44] *p*-values ≤0.05) and presented a class probability >0.9. Each identified SNV was validated by targeted ultra-deep re-sequencing (>1000X). The grey line indicates the expected VAF (50%) for germline or clonal somatic heterozygous variants. *Dark cyan* squares, *grey dot*s and *white* diamonds represent validated somatic, germline and non-validated variations respectively

We then evaluated our trained Model 1A on the remaining data from Dataset 1 that consisted of 34 B- and T-cell cALL patients. To identify somatic variations with high driver potential, only predicted deleterious SNVs with Sift [44] *p*-values ≤0.05 were considered. 50 heterozygous candidate SNVs (VAF <0.6) presenting a class probability over 0.9 and a coverage of at least 15X in the normal sample were randomly selected for validation. These variations showed coverage values ranging from 23 to 115X (mean coverage 51X) and VAFs ranging from 0.10 to 0.57 (median 0.38). For orthogonal validation of this dataset, we used targeted ultra-deep sequencing (Illumina) of the patient’s tumor material (>1000X) and of the normal counterpart in order to confirm the somatic nature of each of the identified variants (Fig. 6, Additional file 2).

A total of 90% (45/50) of the tested SNVs were confirmed real variants, that is found in the tumor material of the patient following our filtering criteria (see Methods). Among these 45 variations, 80% (36/45) were validated somatic mutations (present in tumor only) and 20% (9/45) were identified as germline. Overall, if the confirmed somatic variations are considered true positives and the errors (no calls in re-sequencing) combined with germline variations are considered as false positives, SNooPer’s somatic SNV identification reached a precision of 0.71 (see Methods). As expected, the identified germline mutations had VAFs around the expected clonal heterozygous allele frequency of 0.50 with a mean VAF of 0.47 and a variance (σ^*2*^) of 0.004, while the confirmed somatic mutations had a lower mean VAF of 0.36 associated with a wider distribution (σ^*2*^ = 0.009) averaged from the different subclones present in the sample. Importantly, SNooPer showed no bias of performance in calling subclonal SNVs with low VAF with 2 FPs under and 3 FPs over the median VAF of 0.36, and reached a precision of 0.90 for mutations located within the lower 50^th^ percentile.

## Conclusion

Most available somatic SNV calling methods offer user-defined categorical filters or at best, numerical filters to fine-tune or customize SNV calling, however these can have a strong influence on the output. SNooPer does not rely on user-defined parameters and in doing so, allows versatility and flexibility to cope with complex datasets. Here, the model is directly built around the data itself therefore limiting any bias or subjectivity in somatic mutation calling. Firstly, although systematic errors in the training dataset are likely to exist, the use of an independently sequenced (different technology, mapping) validation dataset will teach SNooPer to recognize systematic errors from the original dataset and to classify them as false positive. Therefore, this method leads to a by-default elimination of systematic errors associated to each sequencing platform. Furthermore, rather than using standardized filters, the importance of each feature for variant classification is directly measured from the data. While any RF algorithm includes by default attribute selection, we also provided the possibility for users to perform a dimensionality reduction of features based on information gain. In doing so we reduce the chance of false positive occurrence due to a strong yet biased feature, which may, in part, explain SNooPer’s superior performance compared to other tested callers. Moreover, SNooPer can accommodate reduced training datasets, such as the one constituted of 250 false and true positives used here, compensate the balance bias using cost sensitive training, and still outperform other commonly used somatic variant callers. Although not reported here, SNooPer also offers an Indel training algorithm and the corresponding calling option that is available in the latest released version. Finally, given that sequencing errors have been linked to homopolymers or G-rich sequence motifs, an updated version of the software that considers the context of genomic coordinates is under development.

As NGS moves toward the clinic and proves its usefulness as a powerful diagnostic tool, whole-genome approaches remain limited to rapid low-pass whole-genome sequencing as a cost-compatible compromise. Sensitive calling algorithms such as SNooPer that is tailored around the data, will thus be indispensable to weed out true somatic variants and identify potential driver mutations or actionable targets. SNooPer was developed in response to this need and has already proven its utility in identifying novel mutations in childhood leukemia [45–47].

## Availability and requirements

**Project name:** SNooPer

**Project home page:** http://www.somaticsnooper.com/, https://sourceforge.net/projects/snooper/

**Operating systems:** any operating system supporting Perl (v5.18.2 or greater) and Java Runtime Environment (v1.5 or greater)

**Programming language:** Perl

**Other requirements:**

- Weka: the published version of SNooPer was tested using the version weka-3-6-10

- R: the published version of SNooPer was tested using the version R/3.2.1

- additional Perl modules: Math::CDF, Text::NSP::Measures::2D::Fisher, Statistics::Test::WilcoxonRankSum and Statistics::R

- Bedtools (optional: if BlackList (-r) or germDB_track (-g) options are applied): the published version of SNooPer was tested with version bedtools-2.17.0

**License:** GNU GPL-3

**Any restriction to use by non-academics:** none

## Additional files

Additional file 1: Table S1. List of SNooPer’s features and descriptions. (PDF 118 kb)
Additional file 2: Supplementary information on datasets, models, comparison with other methods, real data analysis, feature selection, installation and usage. (DOCX 20 kb)
Additional file 3: Figure S1. Snapshot of a SNooPer output from the training phase. (PDF 1143 kb)
Additional file 4: Figure S2. Distribution of VAFs called by SNooPer (Model 1A), MuTect, JointSNVMix and Varscan2 on the test set. (PDF 1469 kb)

## Abbreviations

AUC: Areas under the curve
BQV: Base quality value
cALL: Childhood acute lymphoblastic leukemia
FP: False positive
IG: Information gain
Indel: Insertion and deletion
MQV: Mapping quality value
NGS: Next-generation sequencing
Oob error: Out-of-bag error
PR: Precision-recall
RF: Random forest
ROC: Receiver operating characteristic
SNV: Single nucleotide variant
TP: True positive
VAF: Variant allele frequency
WES: Whole-exome sequencing
WGS: Whole-genome sequencing.

## References

[1] Bonilla X, Parmentier L, King B, Bezrukov F, Kaya G, Zoete V (2016). et aI. Genomic analysis identifies new drivers and progression pathways in skin basal cell carcinoma. Nat Genet.

[2] Krauthammer M, Kong Y, Bacchiocchi A, Evans P, Pornputtapong N, Wu C (2015). Exome sequencing identifies recurrent mutations in NF1 and RASopathy genes in sun-exposed melanomas. Nat Genet.

[3] Al-Ahmadie HA, Iyer G, Lee BH, Scott SN, Mehra R, Bagrodia A (2016). Frequent somatic CDH1 loss-of-function mutations in plasmacytoid variant bladder cancer. Nat Genet.

[4] Barbieri CE, Baca SC, Lawrence MS, Demichelis F, Blattner M, Theurillat JP (2012). Exome sequencing identifies recurrent SPOP, FOXA1 and MED12 mutations in prostate cancer. Nat Genet.

[5] Grasso CS, Wu YM, Robinson DR, Cao X, Dhanasekaran SM, Khan AP (2012). The mutational landscape of lethal castration-resistant prostate cancer. Nature.

[6] Giannakis M, Hodis E, Jasmine Mu X, Yamauchi M, Rosenbluh J, Cibulskis K (2014). RNF43 is frequently mutated in colorectal and endometrial cancers. Nat Genet.

[7] Tan J, Ong CK, Lim WK, Ng CC, Thike AA, Ng LM (2015). Genomic landscapes of breast fibroepithelial tumors. Nat Genet.

[8] Lim WK, Ong CK, Tan J, Thike AA, Ng CC, Rajasegaran V (2014). Exome sequencing identifies highly recurrent MED12 somatic mutations in breast fibroadenoma. Nat Genet.

[9] Shah SP, Roth A, Goya R, Oloumi A, Ha G, Zhao Y (2012). The clonal and mutational evolution spectrum of primary triple-negative breast cancers. Nature.

[10] Ellis MJ, Ding L, Shen D, Luo J, Suman VJ, Wallis JW (2012). Whole-genome analysis informs breast cancer response to aromatase inhibition. Nature.

[11] Stephens PJ, Tarpey PS, Davies H, Van Loo P, Greenman C, Wedge DC (2012). The landscape of cancer genes and mutational processes in breast cancer. Nature.

[12] Banerji S, Cibulskis K, Rangel-Escareno C, Brown KK, Carter SL, Frederick AM (2012). Sequence analysis of mutations and translocations across breast cancer subtypes. Nature.

[13] Rausch T, Jones DT, Zapatka M, Stütz AM, Zichner T, Weischenfeldt J (2012). Genome sequencing of pediatric medulloblastoma links catastrophic DNA rearrangements with TP53 mutations. Cell.

[14] Kataoka K, Nagata Y, Kitanaka A, Shiraishi Y, Shimamura T, Yasunaga J (2015). Integrated molecular analysis of adult T cell leukemia/lymphoma. Nat Genet.

[15] Choi J, Goh G, Walradt T, Hong BS, Bunick CG, Chen K (2015). Genomic landscape of cutaneous T cell lymphoma. Nat Genet.

[16] De Keersmaecker K, Atak ZK, Li N, Vicente C, Patchett S, Girardi T (2013). Exome sequencing identifies mutation in CNOT3 and ribosomal genes RPL5 and RPL10 in T-cell acute lymphoblastic leukemia. Nat Genet.

[17] Holmfeldt L, Wei L, Diaz-Flores E, Walsh M, Zhang J, Ding L (2013). The genomic landscape of hypodiploid acute lymphoblastic leukemia. Nat Genet.

[18] Quesada V, Conde L, Villamor N, Ordóñez GR, Jares P, Bassaganyas L (2011). Exome sequencing identifies recurrent mutations of the splicing factor SF3B1 gene in chronic lymphocytic leukemia. Nat Genet.

[19] Burrell RA, McGranahan N, Bartek J, Swanton C (2013). The causes and consequences of genetic heterogeneity in cancer evolution. Nature.

[20] Xu H, DiCarlo J, Satya RV, Peng Q, Wang Y (2014). Comparison of somatic mutation calling methods in amplicon and whole exome sequence data. BMC Genomics.

[21] Ma X, Edmonson M, Yergeau D, Muzny DM, Hampton OA, Rusch M (2015). Rise and fall of subclones from diagnosis to relapse in pediatric B-acute lymphoblastic leukaemia. Nat Commun.

[22] Landau DA, Carter SL, Stojanov P, McKenna A, Stevenson K, Lawrence MS (2013). Evolution and impact of subclonal mutations in chronic lymphocytic leukemia. Cell.

[23] Green MR, Gentles AJ, Nair RV, Irish JM, Kihira S, Liu CL (2013). Hierarchy in somatic mutations arising during genomic evolution and progression of follicular lymphoma. Blood.

[24] Welch JS, Ley TJ, Link DC, Miller CA, Larson DE, Koboldt DC (2012). The origin and evolution of mutations in acute myeloid leukemia. Cell.

[25] Mullighan CG, Phillips LA, Su X, Ma J, Miller CB, Shurtleff SA (2008). Genomic analysis of the clonal origins of relapsed acute lymphoblastic leukemia. Science.

[26] Landau DA, Carter SL, Getz G, Wu CJ (2014). Clonal evolution in hematological malignancies and therapeutic implications. Leukemia.

[27] Koboldt DC, Zhang Q, Larson DE, Shen D, McLellan MD, Lin L (2012). VarScan 2: somatic mutation and copy number alteration discovery in cancer by exome sequencing. Genome Res.

[28] Larson DE, Harris CC, Chen K, Koboldt DC, Abbott TE, Dooling DJ (2011). SomaticSniper: identification of somatic point mutations in whole genome sequencing data. Bioinformatics.

[29] Roth A, Ding J, Morin R, Crisan A, Ha G, Giuliany R (2012). JointSNVMix: a probabilistic model for accurate detection of somatic mutations in normal/tumour paired next-generation sequencing data. Bioinformatics.

[30] Saunders CT, Wong WS, Swamy S, Becq J, Murray LJ, Cheetham RK (2012). Strelka: accurate somatic small-variant calling from sequenced tumor-normal sample pairs. Bioinformatics.

[31] Cibulskis K, Lawrence MS, Carter SL, Sivachenko A, Jaffe D, Sougnez C (2013). Sensitive detection of somatic point mutations in impure and heterogeneous cancer samples. Nat Biotechnol.

[32] Wang Q, Jia P, Li F, Chen H, Ji H, Hucks D (2013). Detecting somatic point mutations in cancer genome sequencing data: a comparison of mutation callers. Genome Med.

[33] Breiman L (2001). Random Forests. Achine Learning.

[34] Kullback S (1959). Information theory and statistics.

[35] Hall M, Frank E, Holmes G, Pfahringer B, Reutemann P, Witten IH. Data mining in bioinformatics using Weka. Bioinformatics. 2004;20(15):2479–81. 10.1093/bioinformatics/bth261 15073010

[36] Quinlan AR. BEDTools: The Swiss-Army Tool for Genome Feature Analysis. Curr Protoc Bioinformatics. 2014;47:11.12.1–11.12.34. 10.1002/0471250953.bi1112s47 PMC4213956 25199790

[37] Abecasis GR, Auton A, Brooks LD, DePristo MA, Durbin RM, 1000 Genomes Project Consortium (2012). An integrated map of genetic variation from 1,092 human genomes. Nature.

[38] UCSC. UCSC Genome Informatics Group. 2016. [cited 17 July 2016]. Available: https://genome.ucsc.edu/

[39] Healy J, Bélanger H, Beaulieu P, Larivière M, Labuda D, Sinnett D (2007). Promoter SNPs in G1/S checkpoint regulators and their impact on the susceptibility to childhood leukemia. Blood.

[40] Baccichet A, Qualman SK, Sinnett D (1997). Allelic loss in childhood acute lymphoblastic leukemia. Leuk Res.

[41] Cohen J (1960). A coefficient of agreement for nominal scales. Educ Psychol Meas.

[42] Quinlan JR (1993). Morgan Kaufmann Publishers Inc..

[43] Li H, Handsaker B, Wysoker A, Fennell T, Ruan J, Homer N (2009). The Sequence alignment/map (SAM) format and SAMtools. Bioinformatics.

[44] Ng PC, Henikoffa S (2003). SIFT: predicting amino acid changes that affect protein function. Nucleic Acids Res.

[45] Spinella JF, Healy J, Saillour V, Richer C, Cassart P, Ouimet M (2015). Whole-exome sequencing of a rare case of familial childhood acute lymphoblastic leukemia reveals putative predisposing mutations in Fanconi anemia genes. BMC Cancer.

[46] Spinella JF, Cassart P, Garnier N, Rousseau P, Drullion C, Richer C (2015). A novel somatic mutation in ACD induces telomere lengthening and apoptosis resistance in leukemia cells. BMC Cancer.

[47] Spinella JF, Cassart P, Richer C, Saillour V, Ouimet M, Langlois S (2016). Genomic characterization of pediatric T-cell acute lymphoblastic leukemia reveals novel recurrent driver mutations. Oncotarget.

