# Supplementary material for: SNooPer: a machine learning-based method for somatic variant identification from low-pass next-generation sequencing
Source: BMC Genomics. 2016 Nov 14;17:912. doi: 10.1186/s12864-016-3281-2 (PMC5109690; doi:10.1186/s12864-016-3281-2)
Supplement: Additional file 1: Table S1. — List of SNooPer’s features and descriptions. (PDF 118 kb) [file 12864_2016_3281_MOESM1_ESM.pdf]

Additional File 1 (S1 Table). List of SNooPer's features and descriptions.

Group	Feature	Description	IG (model 1A)
Coverage and VAF	allelic_freq_highqual	High quality VAF (BQV > 20, MQV > 10)	0.04187
	highqualcoverage_vs_med	Number of high quality reads (MQV >10) supporting the alternative bases normalized by the median value	0.00940
Location along the read	LocMean_vs_med	Mean location of alternative bases on the reads normalized by the median value	0.00130
	LocMean_vs_ref	Ratio of the mean location of alternative bases on the reads over the mean location of reference bases on the reads	0.00000
Quality bias of alternative bases	GQBprob	Wilcoxon rank-sum test comparing BQVs of reference and alternative bases (p-value)	0.01423
	var_mean_mqv_quality	Mean MQV of alternative bases (Phred score)	0.01210
	var_mean_bqv_quality	Mean BQV of alternative bases (Phred score)	0.00883
	QBpval	Fisher's exact test comparing the number of reference and alternative bases matching the quality criteria (p-value)	0.00852
	RQBprob	Wilcoxon rank-sum test comparing MQVs of reference and alternative bases (p-value)	0.00641
	bqv_ratio_vs_med	Ratio of mean BQV of alternative bases over mean BQV of reference bases normalized by the median value	0.00559
	mqv_ratio_vs_med	Ratio of mean MQV of alternative bases over mean MQV of reference bases normalized by the median value	0.00156
Strand bias	SBpbinom	Probability of having n reads on the less represented strand for a coverage of N knowing that each reads has a probability of 0.5 to be aligned on each strand (cumulative probability from the Binomial distribution)	0.00224
	FRratio	Ratio of the number of reads supporting the alternative bases over the number of reads supporting the reference bases	0.00221
	SBpval	Fisher's exact test comparing the repartion of reference and alternative bases on positive and negative strands (p-value)	0.00103
Other	indelflag*	Flag indicating the presence of indels aligned at the considered position	0.00000

As an example, the information gain (IG) of each feature was measured with respect to the class (InfoGainAttributeEval method, Weka suite) for Model 1A and is indicated in the last column of the table. Features presenting the suffix "vs_med" were normalized using a median value calculated from variants randomly extracted from mpileups files. Features presenting the suffix "vs_ref" were evaluated with respect to reference bases at the same position. (*) Available feature but not used to construct SNooPer's Model 1A.
