# Supplementary material for: SNooPer: a machine learning-based method for somatic variant identification from low-pass next-generation sequencing
Source: BMC Genomics. 2016 Nov 14;17:912. doi: 10.1186/s12864-016-3281-2 (PMC5109690; doi:10.1186/s12864-016-3281-2)
Supplement: Additional file 2: — Supplementary information on datasets, models, comparison with other methods, real data analysis, feature selection, installation and usage. (DOCX 20 kb) [file 12864_2016_3281_MOESM2_ESM.docx]

**SNooPer: a machine learning-based method for somatic variant identification from low-pass next-generation sequencing.**

**Jean-François Spinella^1^, Pamela Mehanna^1^, Ramon Vidal^1^, Virginie Saillour^1^, Pauline Cassart^1^, Chantal Richer^1^, Manon Ouimet^1^, Jasmine Healy^1^, Daniel Sinnett^1,2^**

**1) CHU Sainte-Justine Research Center, Université de Montréal, Montreal, Qc, Canada; 2) Department of Pediatrics, Faculty of Medicine, Université de Montréal, Montreal, Qc, Canada.**

***Supplementary Information***

***Datasets***

**Dataset 1** consisted of 80 cALL patient exomes (40 tumor and 40 normal) (Fig 2 and S2 Table). Whole exomes were captured in solution with Agilent’s SureSelect Human All Exon (50Mb) kit according to the manufacturer’s protocol and sequenced on the Life Technologies SOLiD 4 System (mean coverage on targeted region =30X) at the Integrated Clinical Genomic Centre in Pediatrics, CHU Sainte-Justine. Reads were aligned to the Hg19 reference genome using LifeScope Genomic Analysis Software. PCR duplicates were removed using Picard [1]. Genotype quality score recalibration was performed using the Genome Analysis ToolKit (GATK) [2]. After filtering out low quality reads, mpileup files were created from BAM files using SAMtools [3] (Fig 1).

**Dataset 2** consisted of a subset of 12 cALL patient genomes (6 tumor and 6 normal) overlapping Dataset 1 (Fig 2 and S2 Table). Whole genomes were sequenced by Illumina, Inc. on the HiSeq 2000 (mean coverage =90X); resulting reads were aligned to the Hg19 reference genome using the Illumina Casava software. Bam files were cleaned and mpileup files created as above. SNVs were called from cleaned BAM files using GATK HaplotypeCaller [2] and filtered according to the Broad Institute recommendations (QD <2.0, MQ <40.0, FS >60.0, MQRankSum <-12.5 and ReadPosRankSum <-8.0).

**Dataset 3** was composed of 2 samples sequenced at higher depth on the Illumina system and overlapping Datasets 1 and 2 (Fig 2 and S2 Table). Here, exomes were captured using Illumina’s Nextera Exome Enrichment kit following the manufacturers’ protocol and sequenced on the HiSeq 2500 at the Integrated Clinical Genomic Centre in Pediatrics, CHU Sainte-Justine with a mean coverage of 200X. Mapping to the Hg19 reference genome was performed using Bowtie2 [4] and BAM files were cleaned and mpileup files created as above. SNVs were called using GATK HaplotypeCaller as described for Dataset 2.

The sequencing quality reports are available upon request. For all 3 Datasets, the genomic regions considered for further analysis and comparison were limited to the NCBI's Reference Sequence (RefSeq) [5].

***Models***

To train the 4 distinct models used here (model 1A, 1B, 1C and 2), we considered the GATK HaplotypeCaller [2] output of Dataset 2 as the orthogonal validation of Dataset 1 (Fig 2). Although datasets 2 and 3 shared similar sequencing technologies (Illumina) and were therefore not orthogonal, given the differences of chemistry, platforms, coverage and mapping processes, we considered Dataset 3 to be a reliable validation set for Dataset 2. To construct models 1A, 1B and 2, 30,000 positions presenting alternative bases in the mpileup files of the test Datasets (1 or 2) and not identified by HaplotypeCaller [2] in the validation Datasets (2 or 3) were randomly selected and considered as false positives. Conversely, 250 overlapping mutations between validation and test sets were considered as true positives. 300 trees were used to construct models 1A and 2 and 1,000 trees for model 1B. To construct model 1C, a balanced training set consisting of 250 false and 250 true positives was used. For all our analyses, we performed paired normal/tumor somatic analysis and further filtered out variants that overlapped with 1000 Genomes (2012) [6]. Furthermore, overlaps with the RepeatMasker sequence obtained from UCSC genome browser [7] were excluded to avoid putative miscalled variants located in repetitive elements, including low-complexity sequences and interspersed repeats. To determine the optimal number of trees to be generated, we gradually increased this value (from 100 to 1,000) and determined that, as the number of trees grew beyond 300, classifier performance was only slightly increased at the expense of processing time. Therefore, the default number of trees for SNooPer's RF was set to 300.

***Comparison with other methods***

Given that classes (TP or FP somatic SNV calls) used to train SNooPer's RF model were based on the GATK HaplotypeCaller analysis of Dataset 2, and in order not to favor SNooPer over the other algorithms tested, we used an independent somatic mutation caller (Strelka [8]) for somatic SNV analysis in the test dataset. Overlapping mutations between Datasets 1 and 2 with a VAF >0.10 in Dataset 1 and confirmed as somatic by Strelka in Dataset 2 were considered as true positives; overlapping non-somatic mutations were omitted. Conversely, variant positions in Dataset 1 that were not identified in Dataset 2 were considered as false positives. Positions matching the described criteria were retained in the original Bam files for further assessment by SNooPer and comparison with other methods.

Using this dataset, we compared SNooPer to 3 benchmarked somatic SNV callers: i) Varscan2 (version 2.3.6) [9] was run in somatic mode using a pipe from SAMtools mpileup with a minimum mapping quality value (minBaseQ) of 10, the strand bias filter turned on (strand-filter = 1), a minimum of tumor (min-coverage-tumor) and normal coverage (min-coverage-normal) of 10; ii) JointSNVMix and JointSNVMix2 (version 0.7.5) [10] were first trained ('train' mode) with default parameters (including 'min_normal_depth' and a 'min_tumour_depth' of 10) to tune the parameters and were then run in 'classify' mode. Optional parameters 'minimum base quality' (min_base_qual) and 'minimum mapping quality' (min_map_qual) were set to 20 and 10, respectively. JointSNVMix yielded more accurate results in terms of sensitivity/specificity and was therefore used in our comparative analysis; we did not consider JointSNVMix2 any further; iii) MuTect (version 1.1.4) was run in high confidence (HC) mode with COSMIC version 54 [11] and dbSNP132 [12] as input. We also ran MuTect in ''artifact-detection-mode'', which increased sensitivity at the expense of specificity, therefore this option was not considered for the comparative analysis.

SNooPer performance was assessed and compared to the performance of each benchmarked method by measuring the false discovery rate (FDR), as well as the precision, defined as the ratio of the number of real variants retrieved (TP) to the total number of real variants and errors retrieved (TP+FP), and the recall, defined as the ratio of the number of real variants retrieved (TP) to the total number of real variants in the dataset (TP+FN):


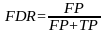

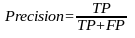

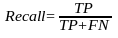


For SNooPer, we kept somatic SNVs (p-value =0) flagged as ”PASS” and varied the class probabilities (from 0.537 to 0.987, 0.519 to 0.949 and 0.756 to 0.930 for models 1A, 1B and 1C, respectively). For Varscan2, only variants identified as “somatic” were considered and we evaluated the output based on somatic p-value that varied from 0.1669 to 3.2577E-8. JointSNVMix analysis was evaluated based on different somatic P-values that varied from 0.2901 to 1. Finally, we considered mutations annotated as “KEEP” and “COVERED” by MuTect and varied the “power” (from 0.1614 to 0.9994). Mutect is very restrictive on its somatic SNV calls and selection of variants annotated as “NOVEL” only resulted in undercalling of true somatic variants.

***Real data analysis***

To assess SNooPer’s performance on a real dataset, we called somatic SNVs on a dataset consisting of 34 cALL patient exomes (68 matched normal-tumor samples) that were sequenced as in Dataset 1 (above). For orthogonal validation, somatic SNVs identified using SNooPer were then subjected to ultra-deep targeted resequencing using Illumina TruSeq Custom Amplicon assay as per the manufacturer’s instructions. Illumina DesignStudio was used to design custom oligos targeting 50 randomly selected high confidence SNVs called by SNooPer. PCR purification was performed with Ampure Beads for 150bp amplicon size selection. Double stranded amplicons were pooled, quantified by qPCR and sequenced on the Illumina HiSeq 2500 system (paired-end: 2x100bp) to reach a minimum of 1000X coverage. Sequenced reads were aligned to the Hg19 reference genome using Bowtie2 [4] and variants were called using Varscan2 ''mpileuptosnp'' analysis [9]. Somatic SNVs identified in tumor only were considered as validated, while variants present in both tumor and normal were considered germline.

***Feature Selection***

Features calculated from Dataset 1 were ranked according to their IG (S1 Table). Different threshold values of IG were measured to filter out less informative features and best results were obtained by eliminating features with less than 0.001 bits of IG. For this dataset, 3 features presented more than 0.01 bits of IG: high quality VAF (allelic_freq_highqual), IG =0.0419; p-value obtained from a Wilcoxon rank-sum test comparing the base quality value (BQV) of reference bases versus alternative bases (GQBprob), IG =0.0142; mean mapping quality value (MQV) of alternative bases (var_mean_mqv_quality), IG =0.0121. Here, VAF was of particular importance for SNooPer’s classification performance: using a wide range of VAFs rather than a binary cutoff led to more efficient calling of subclonal variations (see below). Interestingly, the first feature belonging to the strand bias subgroup (Sbpbinom) only ranked 9th with an IG of 0.0022. This highlighted the limited importance of the strand bias for classification of these data. This could be explained by limited sampling (low coverage) on captured sequences known for their tendency towards artificial strand biases. Given the importance of strand bias in other algorithms tested here, this could lead to misinterpretation of certain performance comparisons.

***Installation and Usage***

INSTALLATION

To install this module, run the following commands:

perl Makefile.pl

make

make test

make install

SYNOPSIS

>SNooPer.pl -help [brief help message] -man [full documentation]

>Training:

>SNooPer.pl -i [input_directory] -o [output_directory] -a1 [type_of_analysis1] -a2 [train] -w [path_to_weka] [options]

>Classify/Evaluate:

>SNooPer.pl -i [input_directory] -o [output_directory] -a1 [type_of_analysis1] -a2 [classify/evaluate] -m [model] -w [path_to_weka] [options]

DESCRIPTION

>SNooPer requires a training phase during which a training dataset (a subset of validated positions) is used to construct a model that can be then applied to call variants on an extended test dataset.

>For the training phase ("train"), the user must provide 2 types of files:

>1.pileup files (.pu) with similar characteristics as the test dataset on which the trained model will be applied.

>Somatic analysis format: tset_T_sample_id.pu and tset_N_sample_id.pu

>Germline analysis format: tset_sample_id.pu

>2.vcf files (.vcf) validation files that are ideally orthogonal validations of the positions contained in the pileup files.

>Somatic analysis format: vset_T_sample_id.vcf

>Germline analysis format: vset_sample_id.vcf

>Each position in the pileup files must be tested a priori so that the class (true variant or sequencing error) is known by comparison with the vcf files.

If a variant is present in the corresponding validation file, it will be considered as an actual variant. If the variant is absent from the validation file, the variant will be considered as an error.

>To be considered as the corresponding validation file of a .pu file, the .vcf file has to present the same sample_id.

>For the classification phase ("classify") or to evaluate a model ("evaluate"), the user simply provides the paths to the model that is to be applied and to the pileup files from the test dataset:

>Somatic analysis format: tset_T_sample_id.pu and tset_N_sample_id.pu

>Germline analysis format: tset_sample_id.pu

>Note that input files must contain the prefix tset_ (for training or test dataset, depending on the context) and the .pu extension or vset_ (for validation dataset) and the .vcf extension.

REQUIREMENTS

>The following programs must be installed:

-Weka; the current version of SNooPer was tested with version weka-3-6-10.

-R; the current version of SNooPer was tested with version R/3.2.1

-Bedtools if BlackList (-r) or germDB_track (-g) options are applied. The current version of SNooPer was tested with version bedtools-2.17.0.

>For the development and testing of SNooPer:

The BlackList track corresponded to the RepeatMasker track downloaded from UCSC. "Assembly" has to be set according to the reference used to map your sequences, "Group" was set to Variation and Repeats, and "Track" was set to RepeatMasker. The track was downloaded in a .bed format.

>The germline database used as germDB_track corresponded to the 1000 Genomes database downloaded from http://www.1000genomes.org/. The track was formated in a .bed format.

OPTIONS

-help <brief help message>

-man <full documentation>

-a1 <type_of_analysis1> Can take the following values: "somatic" or "germline". "somatic" means that the somatic evaluation will be done based on N samples provided (and additional germline data if provided, see germDB_track -g option).

-a2 <type_of_analysis2> Can take the following values: "train", "classify" or "evaluate".

->if "train" is selected, a model will be trained based on the comparison of the training dataset (tset) and the validation dataset (vset). A subset of the data provided (subset chosen with the -v and -nv options or automatically selected) for which the class is known (0/1 = non-validated/validated = not shared by tset and vset / shared by tset and vset) will be used for training. Therefore, a partially overlapping dataset between tset and vset must be provided. Final classification of the complete data will be done base on the trained model. Furthermore, evaluation of the model will be performed using a subset excluded beforehand.

->if "classify" is selected, the provided test dataset (tset) is classified using a model created previously. This model has to be in an .arff format (see Weka documentation for more info).

->if "evaluate" is selected, the provided dataset (tset) is classified using a model created previously. The purpose of this option is to evaluate a previously created model based on the classification of an independent dataset (never used to train the model). To evaluate the model, the class of each variant in the dataset must be known. Therefore, the data from both tset and vset must be provided. These data should be located in a new directory containing these files only.

-i <input_directory> Complete path to your input directory.

-o <output_directory> Complete path to your output directory (input and output can be located in the same directory).

-m <path_to_model> Complete path to the directory of a previously trained model. This option should be set only if the type of analysis 2 is "classify" or "evaluate".

-w <path_to_weka> Complete path to the weka.jar executable.

--------------------------------------------------------

-a3 <type_of_analysis3> [optional] Can take the following values: "SNP" or "Indel". The default value is "SNP".

-a4 <attributes_selection> [optional] Can take the following value: "off", "MI" or "BestFirst". The default value is "off". If "MI" is selected (Weka InfoGainAttributeEval + Ranker): evaluation the worth of an attribute by measuring the information gain with respect to the class + ranking of attributes by their individual evaluations. Attributes will be discarded if presenting less than 0.001 bits of mutual information. If "BestFirst" is selected (Weka CfsSubsetEval + BestFirst): evaluate the value of a subset of attributes by considering the individual predictive ability of each feature along with the degree of redundancy between them + evaluate the space of attribute subsets by greedy hillclimbing augmented with a backtracking facility.

-b <path_to_bedtool> [optional] Complete path to bedtools binary file.

-bqv <bqv> Base quality value (phred) of a variation to be considered as "High Quality". Default value is 20.

-c <contamination> [optional] Fraction of normal cells in the tumor sample. Can take a value between 0 and 1. Default value is 0.

-cf <covered_filter_N> [optional] Can take the following values: "on" or "off". If the filter is "on", only positions with a minimum coverage of "coveragefilter_N" in the N will be considered in the T for somatic analysis. Default value is on.

-cm <cost_matrix> [optional] used to adjust the weight of mistakes on a class (see http://weka.wikispaces.com/CostMatrix). The cost matrix has to be define in a single line format using comma to separate values ex: 0.0,5.0,1.0,0.0 here the weight on false positive is 5 and on false negatives is 1.

-cn <coveragefilter_N> [optional] Defines the minimum of coverage for a position to be considered in the N files during a Somatic analysis or the Germline analysis. If a position in the T file doesn't reach the coverage limit in the N file, the position can't be call Somatic and won't be considered. Default value is 8.

-ct <coveragefilter_T> [optional] Defines the minimum coverage required for a position to be considered in the T file during a Somatic analysis. Default value is 8.

-fi <freqinf> [optional] Defines the inferior limit of allele frequency for a variant position to be considered in the T file during a Somatic analysis. Default value is 0.

-fs <freqsup> [optional] Defines the superior limit of allele frequency for a variant position to be considered in the T file during a Somatic analysis. Default value is 1.

-g <path_to_germDB_track> [optional] Complete path to any germline variant database track. If such a file is provided and if the type_of_analysis1 is "somatic", the variations located at these positions will be considered as germline during the somatic variant calling process.

-id <job_id> [optional] The output file name will be: SNooPer_output_job_id_date.

-ind <indel_filter> [optional] Can take the following values: "on" or "off" when type_of_analysis3 is "SNP". If the filter is "on", pileup lines containing indels won't be considered during the SNP calling process. Default value is on.

-k <cross_validation> [optional] Integer to define the k-fold cross-validation used to train the model. This option must be set only if the type of analysis 2 is "train" or "classify". Default value is 10.

-mem <memory> [optional] The user can extend the memory available for the virtual machine by setting appropriate options. Ex: -Xmx2g to set it to 2GB. The user can also redirect temporary JVM files using the format: -Djava.io.tmpdir=/path/to/tmpdir

-mqv <mqv> [optional] Minimum mapping quality value (phred) of a read in order for it to be retained as "High Quality" in the variant calling process. Default value is 20.

-nN <nbvar_N> [optional] Defines the number of supporting variant reads required for a position to be considered in the N files during a Germline or Somatic analysis.

-nT <nbvar_T> [optional] Defines the number of supporting variant reads required for a position to be considered in the T files during a Somatic analysis.

-nv <nb_of_non_validated_var_to_train> [optional] Number of non-validated variants (disconcordant between tset and vset) used to train your model. If no value is provided, a default value will be calculated from the input file. It prevails over validated_variant_fraction and validated_nonvalidated_ratio.

-p1 <tech> [optional] Technology/chemistry used to produce the data to be classified. Can take the following values: "Solid", "Solexa", "Illumina-1.3", "Illumina-1.5" or ">Illumina-1.8". Default value is the Illumina-1.8 or higher ">Illumina-1.8".

-q <qual_filter> [optional] Can take the following values: "on", "on+", "off" or "off". If the filter is "on" or "on+", only variants matching the selected bqv and mqv values will be considered. If "on+" or "off+" are selected, all attributes will be considered including those that depend on quality. Default value is on.

-r <path_to_blacklist> [optional] Complete path to the BlackList track. This black list usually corresponds to problematic regions in the genome. If such a file is provided, the variations located in these regions won't be considered during the variant calling process.

-s <somatic_pvalue> [optional] Somatic P-value filter based on a one-tailed Fisher's exact test comparing the somatic and germline allele count. Only variants presenting a P-value <= to this value will be conserved. The default value is 0.1. The value must be set between 0 and 1.

-t <tree> [optional] Number of trees to build the model. Default value is 300.

-v <nb_of_validated_var_to_train> [optional] Number of validated variants (concordant between tset and vset) used to train your model. If no value is provided, a default value will be calculated from the input file. It prevails over validated_variant_fraction and validated_nonvalidated_ratio.

-vf <validated_variant_fraction> [optional] Fraction of the validated variants to be used for training. The default value is 1. Note that if the number of validated positions is large, the analysis can be time-consuming.

-vr <validated_nonvalidated_ratio> [optional] Ratio (nb of non-validated variants / nb of validated variants) in the training dataset. The default value is 0.1. Note that, if the training dataset is extremely imbalanced, cost sensitive learning can be used to improve the algorithm’s performance.

**References**

1. Picard. Broadinstitute. 2016. [cited 5 Avril 2016]. Available: <http://broadinstitute.github.io/picard/>

2. McKenna A, Hanna M, Banks E, Sivachenko A, Cibulskis K, Kernytsky A, et al. The Genome Analysis Toolkit: a MapReduce framework for analyzing next-generation DNA sequencing data. Genome Res. 2010;20(9): 1297-1303.

3. Li H, Handsaker B, Wysoker A, Fennell T, Ruan J, Homer N, et al. The Sequence alignment/map (SAM) format and SAMtools. Bioinformatics. 2009;25: 2078-2079.

4. Langmead B, Salzberg S. Fast gapped-read alignment with Bowtie 2. Nature Methods. 2012;9: 357-359.

5. RefSeq: Reference Sequence Database. NCBI. 2016 [cited 5 Avril 2016]. Available: <http://www.ncbi.nlm.nih.gov/refseq/>

6. 1000 Genomes Project Consortium, Abecasis GR, Auton A, Brooks LD, DePristo MA, Durbin RM, et al. An integrated map of genetic variation from 1,092 human genomes. Nature. 2012;491(7422): 56-65.

7. UCSC. UCSC Genome Informatics Group. 2016. [cited 5 Avril 2016]. Available: <https://genome.ucsc.edu/>

8. Saunders CT, Wong WS, Swamy S, Becq J, Murray LJ, Cheetham RK. Strelka: accurate somatic small-variant calling from sequenced tumor-normal sample pairs. Bioinformatics. 2012;28(14): 1811-1817.

9. Koboldt DC, Zhang Q, Larson DE, Shen D, McLellan MD, Lin L, et al. VarScan 2: somatic mutation and copy number alteration discovery in cancer by exome sequencing. Genome Res. 2012;22:568-576.

10. Roth A, Ding J, Morin R, Crisan A, Ha G, Giuliany R, et al. JointSNVMix: a probabilistic model for accurate detection of somatic mutations in normal/tumour paired next-generation sequencing data. Bioinformatics. 2012;28: 907-913.

11. Forbes SA, Bhamra G, Bamford S, Dawson E, Kok C, Clements J, et al. The Catalogue of Somatic Mutations in Cancer (COSMIC). Curr Protoc Hum Genet. 2008;Chapter 10: Unit 10.11.

12. dbSNP Short Genetic Variations. NCBI. 2016 [cited 5 Avril 2016]. Available: http://www.ncbi.nlm.nih.gov/SNP/
