# Supplementary material for: SNooPer: a machine learning-based method for somatic variant identification from low-pass next-generation sequencing
Source: BMC Genomics. 2016 Nov 14;17:912. doi: 10.1186/s12864-016-3281-2 (PMC5109690; doi:10.1186/s12864-016-3281-2)
Supplement: Additional file 3: Figure S1. — Snapshot of a SNooPer output from the training phase. (PDF 1143 kb) [file 12864_2016_3281_MOESM3_ESM.pdf]

A

=== Stratified cross-validation ===

Correctly Classified Instances	30175 (99.7521 %)
Incorrectly Classified Instances	75 (0.2479 %)
Kappa statistic	0.8395
Mean absolute error	0.0041
Root mean squared error	0.0429
Relative absolute error	24.9469 %
Root relative squared error	47.4204 %
Total Number of Instances	30250

=== Confusion Matrix ===

a	b	<-- classified as
29977	23	a = 0
52	198	b = 1

B

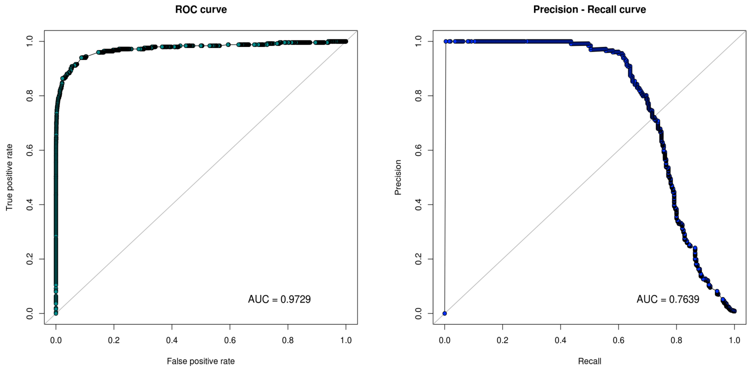

Additional File 3 (S1 Fig). Snapshot of a SNooPer output from the training phase. (**A**) General statistics including Kappa statistics (top) and the confusion matrix (bottom) obtained from a 10-fold cross validation training phase. (**B**) Receiver operating characteristics (left) and precision-recall (right) curves and the related AUCs calculated during the training phase.
