# Supplementary material for: SNooPer: a machine learning-based method for somatic variant identification from low-pass next-generation sequencing
Source: BMC Genomics. 2016 Nov 14;17:912. doi: 10.1186/s12864-016-3281-2 (PMC5109690; doi:10.1186/s12864-016-3281-2)
Supplement: Additional file 4: Figure S2. — Distribution of VAFs called by SNooPer (Model 1A), MuTect, JointSNVMix and Varscan2 on the test set. (PDF 1469 kb) [file 12864_2016_3281_MOESM4_ESM.pdf]

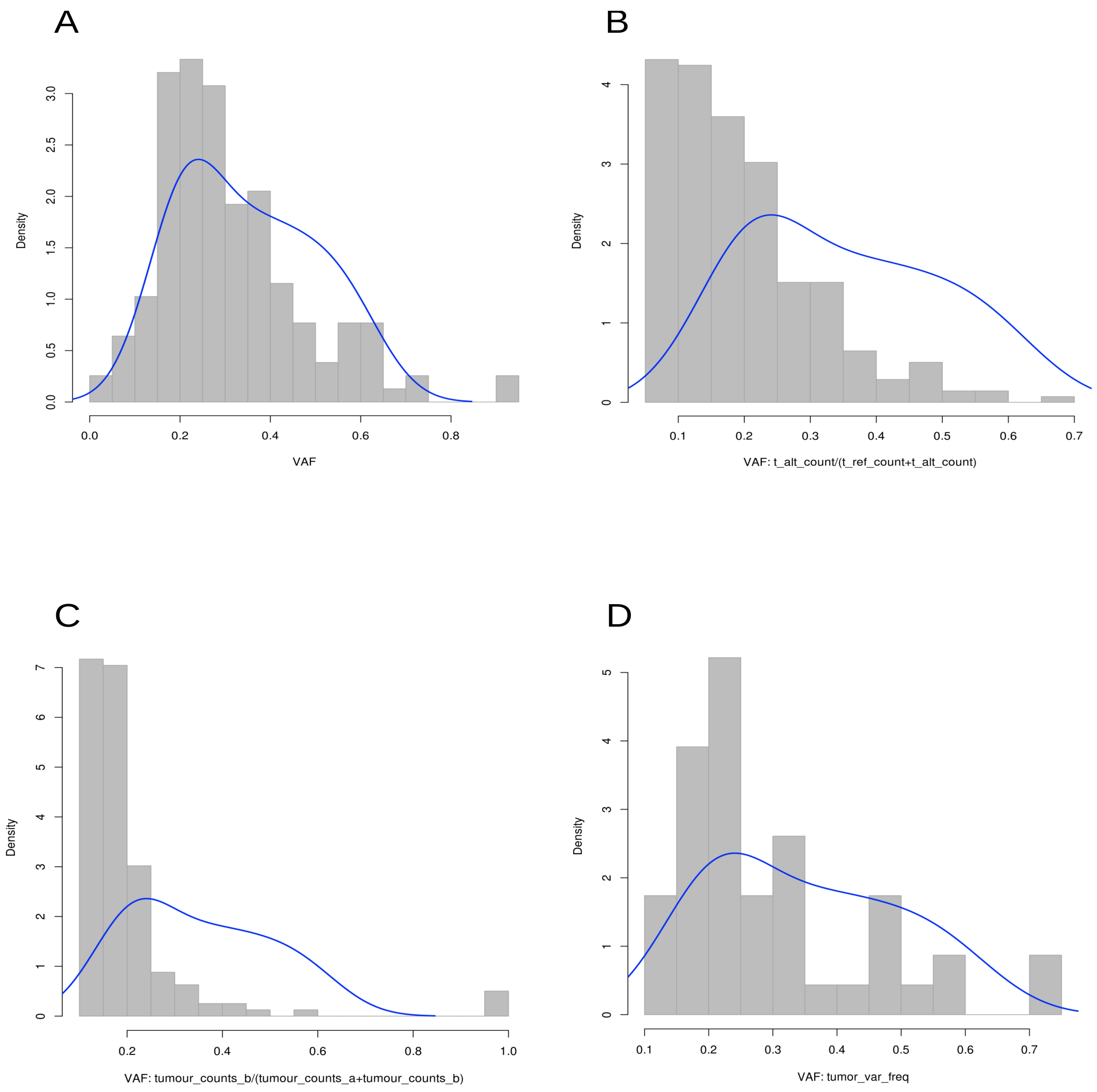

Additional File 4 (S2 Fig). Distribution of VAFs called by (A) SNooPer (Model 1A), (B) MuTect, (C) JointSNVMix and (D) Varscan2 on the test set. Selected somatic SNVs called by SNooPer had to be flagged as "PASS" and to present a class probability >0.5. SNVs called by MuTect had to be flagged as "KEEP" or present a somatic p-value <0.05 for JointSNVMix and Varscan2. In the histogram, bars represent probability densities (relative frequencies) of variants according to VAFs. Blue lines represent the kernel density estimates of true positive variants from the test set.
